# Nomogram forecasting 3‐, 5‐, and 8‐year overall survival and cancer‐specific survival of gingival squamous cell carcinoma

**DOI:** 10.1002/cam4.3436

**Published:** 2020-09-22

**Authors:** Lei Yan, Weizhuo Deng, Lina Guan, Hao Xu

**Affiliations:** ^1^ Department of Oral and Maxillofacial Surgery General Hospital of Xinjiang Military command Urumqi China; ^2^ Department of Stomatology The Affiliated Hospital of Qingdao University Qingdao China; ^3^ School of Stomatology Qingdao University Qingdao China

**Keywords:** calibration curve, gingival squamous cell carcinoma, nomogram, survival analysis

## Abstract

No nomogram models addressing the personalized prognosis evaluation of patients with gingival squamous cell carcinoma (GSCC) have been documented. We sought to establish nomograms to forecast overall survival (OS) and cancer‐specific survival (CSS) of patients with GSCC. We collected the detailed clinicopathological information of 2505 patients with GSCC from the Surveillance, Epidemiology and End Results (SEER) program. Afterward, we divided the 2505 cases into a modeling group (n = 1253) and an external validation cohort (n = 1252) via random split‐sample method. We developed the nomograms on the basis of the Kaplan‐Meier and multivariate Cox survival analysis of the modeling group and then split the modeling cohort into two parts based on cut‐off values: high‐ and low‐risk cohorts. An improved survival was shown in the low‐risk group compared to their counterpart, with a significant difference after the log‐rank test. The performance of the nomograms was evaluated via concordance‐index (C‐index), the area under the receiver operating characteristic curve (AUC), and calibration curves. All the C‐indexes and AUCs were greater than 0.7, showing high accuracy. Moreover, the calibrations showed that the actual observations were close to the 45° perfect reference line. In conclusion, we successfully developed two nomograms to provide individualized, patient‐specific estimates of OS and CSS available for risk‐stratification.

## Graphical abstract

We have successfully established two nomogram models forecasting OS and CSS of GSCC patients. Also, we have validated the model via C‐index, calibration curves and ROC curves.

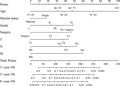

## 1 INTRODUCTION

Oral squamous cell carcinoma (OSCC) located on the tongue, gingival, hard palate, mouth floor, and cheek accounts for 3% all malignant tumors of the body. The incidence and mortality rate of OSCC are different in various regions, commonly occurring in developing countries. GSCC is one of the most familiar malignant tumors among head and neck cancers, constituting 10%‐25% of OSCCs.^1^, ^2^ In terms of etiology, there were many factors that can promote the occurrence and development of GSCC, among which smoking and drinking are the most significant factors.^3^, ^4^ Additionally, the occurrence of malignant lesions could be induced by chronic repeated stimulation and infection, such as poor oral hygiene, residual crown and root, and inappropriate prosthesis.^5^ Maxillary GSCC often invades the palatal mucosa and maxillary sinus invades the infratemporal fossa and pterygopalatine fossa backward, or pierces the nasal cavity, causing epistaxis and increasing nasal secretion.^6^ Mandible GSCC often appears in the posterior teeth area, and invades the mandible along the periosteum to a certain depth.^7^

Over the years, the overall cure and survival rates of patients with tumors have not been significantly improved. The postoperative survival rate of patients with GSCC with recurrence and metastasis is still unsatisfactory. Approximately 28% of patients experience lymph node metastasis (LNM), and the frequency of occult LNM among patients with maxillary GSCC is 27%.^8^, ^9^ Hence, developing a credible model to predict prognosis remains our priority. Notably, the NCCN guidelines suggest evaluating prognosis following the 7th AJCC Staging system.^10^, ^11^ However, a couple of relevant factors might influence the outcome of patients with GSCC, not merely TNM stages.

Nomograms have emerged as an important prediction model to conduct personalized prognosis evaluation. The development of the nomogram is based on the Kaplan‐Meier and Cox regression survival analysis. Notably, the 8th AJCC manual notes that future versions would incorporate nomograms to conduct individualized prognosis assessments. Nomograms have been widely used in numerous fields, such as gastric cancer,^12^ esophageal Cancer,^13^ hepatocellular carcinoma,^14^ colorectal cancer,^15^ and salivary gland cancer.^16^ Most importantly, the NCCN guidelines have incorporated nomograms to aid in the early detection of prostate cancers.^17^ However, no GSCC nomogram prediction models have been documented previously. Hence, for the first time, we attempt to construct nomograms to predict OS and CSS of patients with GSCC.

## 2 PATIENTS AND METHODS

### 2.1 Clinicopathological data

We obtained detailed information of all 2505 patients with GSCC from 2004 to 2013 from the SEER database (http://seer.cancer.gov). We eliminated the cases obtained through autopsies or death certificates. Total patients were randomly divided into the training and validation groups (split‐ratio = 1:1). Patients’ detailed information is noted in Table 1. The definition of OS was a time span ranging from GSCC diagnosis to last follow‐up or death. Moreover, CSS represented the time interval from diagnosis to death owing to GSCC, excluding death due to other reasons.

**TABLE 1:** Patients’ detailed general information

Variables	Training cohort (n = 1253)		Validation cohort ( n = 1252)	
	N	%	N	%
Age				
15‐45	63	5.0	70	5.6
46‐55	153	12.2	181	14.5
56‐65	317	25.3	325	26.0
66‐75	349	27.9	306	24.4
76‐85	253	20.2	263	21.0
85+	118	9.4	107	8.5
Sex				
Male	682	54.4	697	55.7
Female	571	45.6	555	44.3
Site				
Upper	228	18.2	235	18.8
Lower	967	77.2	958	76.5
Other	58	4.6	59	4.7
Race				
White	1086	86.7	1071	85.5
Black	72	5.7	93	7.4
Others	95	7.6	88	7.0
Marital status				
Single	552	44.1	571	45.6
Married	701	55.9	681	54.4
Grade				
I	322	25.7	308	24.6
II	703	56.1	698	55.8
III	223	17.8	238	19.0
IV	5	0.4	8	0.6
Surgery				
Performedd	1053	84.0	1056	84.3
None	200	16.0	196	15.7
Radiation				
Yes	504	40.2	510	59.3
No	749	59.8	742	40.7
T stage				
T1	403	32.2	383	30.6
T2	346	27.6	356	28.4
T3	128	10.2	118	9.4
T4	376	30.0	395	31.5
N stage				
N0	841	67.1	815	65.1
N1	185	14.8	180	14.4
N2	212	16.9	249	19.9
N3	15	1.2	8	0.6
M stage				
M0	1225	97.8	1228	98.1
M1	28	2.2	24	1.9

### 2.2 Survival analysis and nomogram development

We conducted survival analysis via Kaplan‐Meier and Cox regression method using SPSS 21.0 software, which was in accordance with the published literature.^18^ After the above steps, independent prognostic risk factors were obtained and *P* < .05 was deemed as statistically significant. Furthermore, we incorporated the above prognosis‐relevant elements to develop the nomograms via the R software package “cmprsk.”

### 2.3 Nomogram validation procedures

Thousand times bootstrapping and 10‐fold cross‐validation methods were applied to test the nomograms for both the training and validation cohorts internally and externally respectively. C‐index and calibration curves were employed to evaluate the fitting degree of each nomogram.^19^ The calibration plot included a 45° diagonal line and an actual line. The more closer the two lines were, the more accurate was the nomogram. Moreover, the AUC was calculated to evaluate the performance of nomogram.

### 2.4 Patients risk stratification

Nomograms can convert patients’ clinicopathological information into a visual linear graph. We could then calculate each patient's nomogram‐based score. Based on their scores, the training cohort was separated into high‐ and low‐risk groups. We compared the two groups via Kaplan‐Meier survival analysis. *P* < .05 represented a significant difference after the log‐rank test.

## 3 RESULTS

### 3.1 Patients’ general clinicopathological information

After applying a strict filter, 2505 GSCC cases were screened from the SEER database. The training and validation cohorts included 1253 and 1252 cases respectively. Patients’ general clinicopathological information including age, sex, race, marital status, site, grade, radiation, surgery, and TNM stage, is shown in Table 1. Grades I, II, III, and IV represented well differentiated, moderately differentiated, poorly differentiated, and undifferentiated respectively.

The median follow‐up periods for the training and validation groups were 36 and 27 months respectively. In total, the last follow‐up showed that 566 patients were deceased in the training group. Among them, 398 patients died because of GSCC, and 168 patients died of reasons other than GSCC.

### 3.2 Survival analysis and nomogram development

**FIGURE 1:**
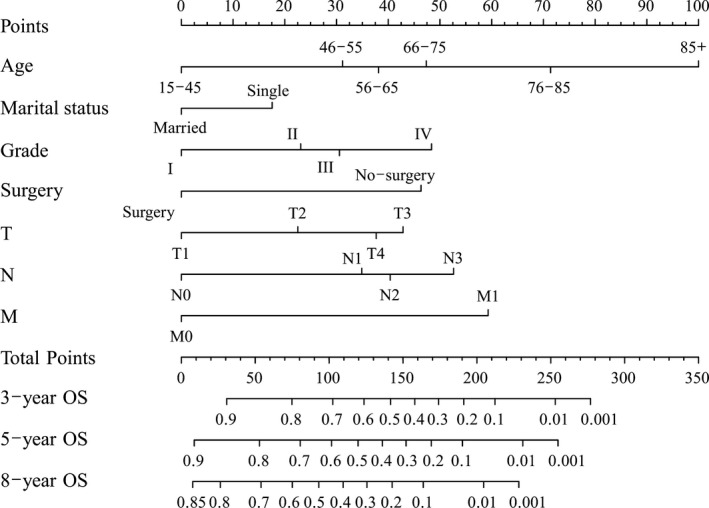
Nomogram predicting overall survival of gingival squamous cell carcinoma patients

The results of OS and CSS analysis are shown in Tables 2 and 3. After performing Kaplan‐Meier univariate OS analysis, we found that age, marital status, site, grade, surgery, T stage, N stage, and M stage were statistically significant (*P* < .05). Furthermore, we incorporated the above elements into multivariate Cox proportional hazards analysis and found that age, marital status, grade, surgery, T stage, N stage, and M stage were independent prognostic indicators (*P* < .05), which are shown in Table 2. Thus, nomograms were developed to predict the 3‐, 5‐ and 8‐year OS rates in the training cohort based on independent prognostic risk factors (Figure 1).

**TABLE 2:** OS analysis regarding training cohort

Variables	Univariate analysis	Multivariate analysis	
	*P‐*value	HR (95% CI)	*P‐*value
Age	<.001		<.001
15‐45		0.188 (0.108‐0.327)	<.001
46‐55		0.317 (0.225‐0.447)	<.001
56‐65		0.356 (0.267‐0.476)	<.001
66‐75		0.416 (0.314‐0.552)	<.001
76‐85		0.623 (0.471‐0.825)	<.001
85+		Reference	
Sex	.540		
Male			
Female			
Site	<.001		.234
Upper		0.930 (0.740‐1.169)	.534
Lower		1.270 (0.843‐1.913)	.253
Other		Reference	
Race	.314		
White			
Black			
Others			
Marital status	<.001		.001
Married		0.743 (0.625‐0.884)	.001
Single		Reference	
Grade	<.001		<.001
I		0.422 (0.132‐1.353)	.147
II		0.632 (0.198‐2.014)	.438
III		0.637 (0.198‐2.048)	.449
IV		Reference	
Surgery	<.001		<.001
Performed		Reference	
None		2.165 (1.765‐2.656)	<.001
Radiation	.450		
Yes			
No			
T stage	<.001		<.001
T1		0.535 (0.423‐0.677)	.147
T2		0.787 (0.635‐0.976)	.438
T3		1.119 (0.859‐1.458)	.449
T4		Reference	
N stage	<.001		<.001
N0		0.409 (0.215‐0.775)	.006
N1		0.737 (0.386‐1.409)	.356
N2		0.811 (0.427‐1.540)	.521
N3		Reference	
M stage	<.001		<.001
M0		0.379 (0.244‐0.589)	<.001
M1		Reference	

**TABLE 3:** CSS analysis regarding training cohort

Variables	Univariate analysis	Multivariate analysis	
	*P‐*value	HR (95% CI)	*P‐*value
Age	<.001		<.001
15‐45		0.336 (0.184‐0.613)	<.001
46‐55		0.372 (0.245‐0.564)	<.001
56‐65		0.452 (0.314‐0.653)	<.001
66‐75		0.545 (0.380‐0.781)	<.001
76‐85		0.807 (0.565‐1.151)	<.001
85+		Reference	
Sex	.269		
Male			
Female			
Site	<.001		.021
Upper		Reference	
Lower		1.004 (0.760‐1.327)	.978
Other		1.732 (1.108‐2.708)	.016
Race	.818		
White			
Black			
Others			
Marital status	<.001		.025
Single		Reference	
Married		0.787 (0.639‐0.970)	.025
Grade	<.001		<.001
I		0.305 (0.129‐0.724)	.007
II		0.429 (0.185‐0.993)	.048
III		0.491 (0.210‐1.146)	.100
IV		Reference	
Surgery	<.001		<.001
Performed		Reference	
None		2.494 (1.973‐3.152)	<.001
Radiation	.208		
Yes			
No			
T stage	<.001		<.001
T1		0.370 (0.276‐0.497)	<.001
T2		0.670 (0.526‐0.854)	.001
T3		0.736 (0.523‐1.037)	.08
T4		Reference	
N stage	<.001		<.001
N0		0.371 (0.155‐0.886)	.026
N1		0.700 (0.293‐1.676)	.423
N2		0.896 (0.376‐2.134)	.803
N3		Reference	
M stage	<.001		.044
M0		0.599 (0.364‐0.986)	.044
M1		Reference	

**FIGURE 2:**
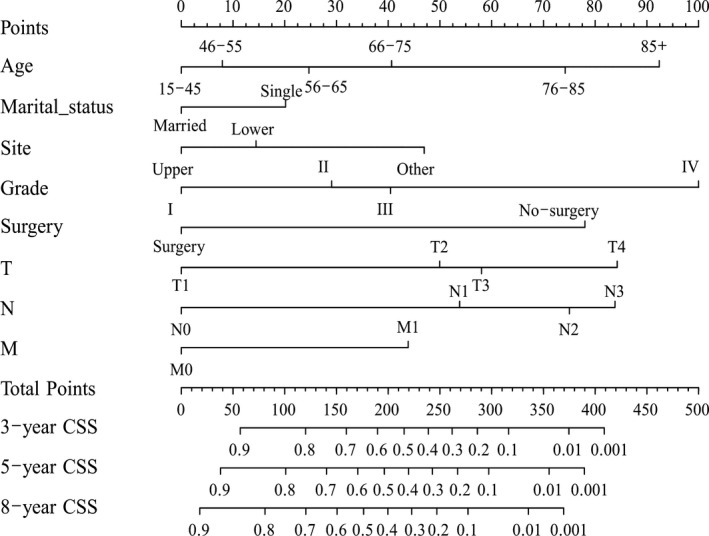
Nomogram predicting cancer‐specific survival of gingival squamous cell carcinoma patients

The results showed that age, marital status, site, grade, surgery, T stage, N stage and M stage were independent prognostic risk factors influencing CSS (Table 3). In addition, we developed another nomogram to predict the CSS of patients with GSCC (Figure 2).

### 3.3 Nomogram validation

**FIGURE 3:**
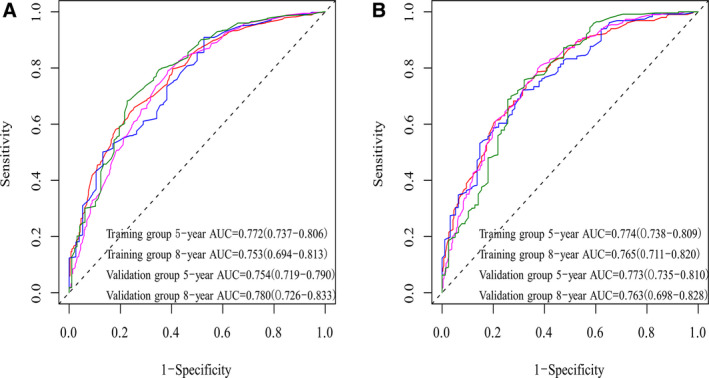
Performance of nomogram via ROC

**FIGURE 4:**
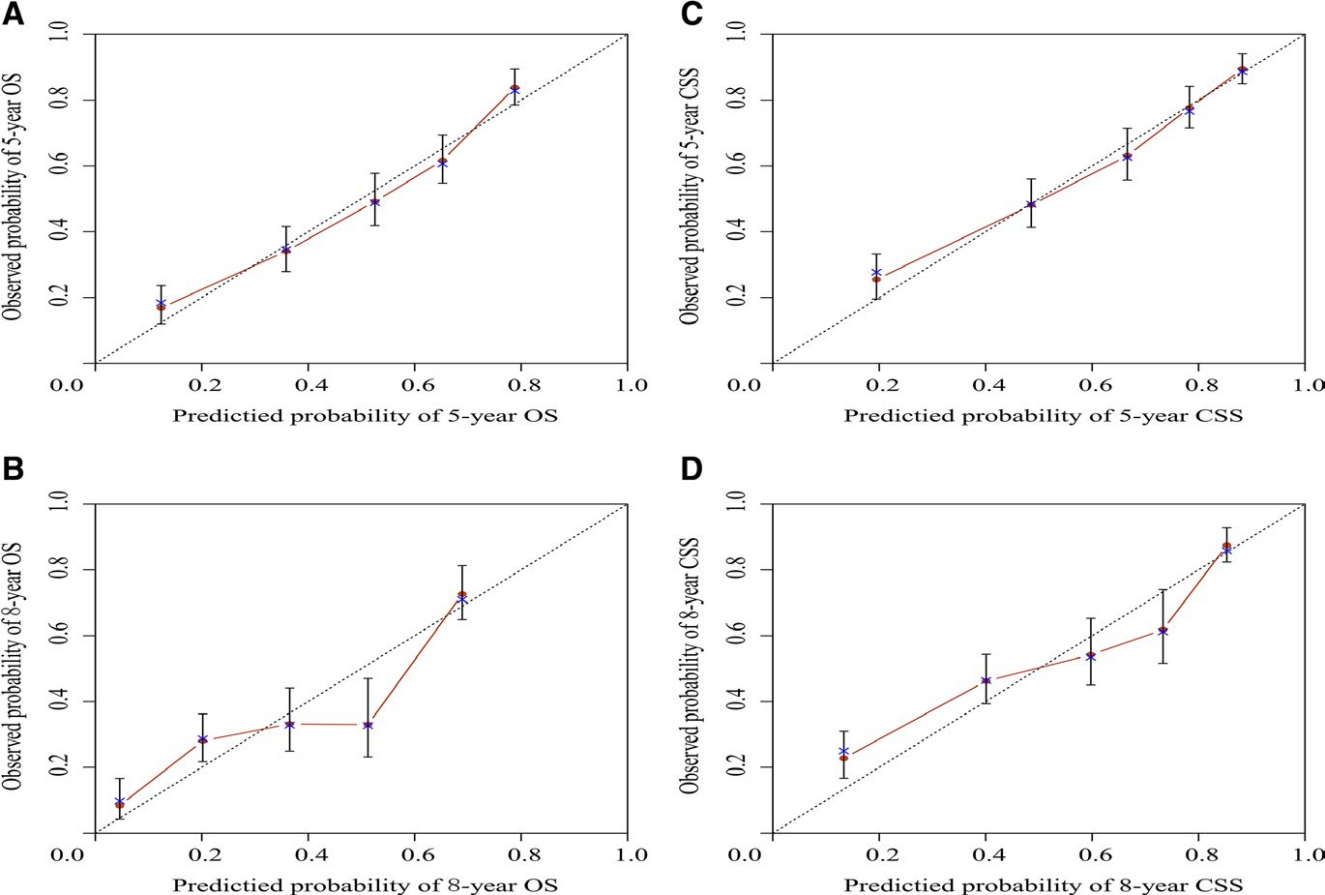
Internal calibration nomogram for OS and cancer‐specific survival

**FIGURE 5:**
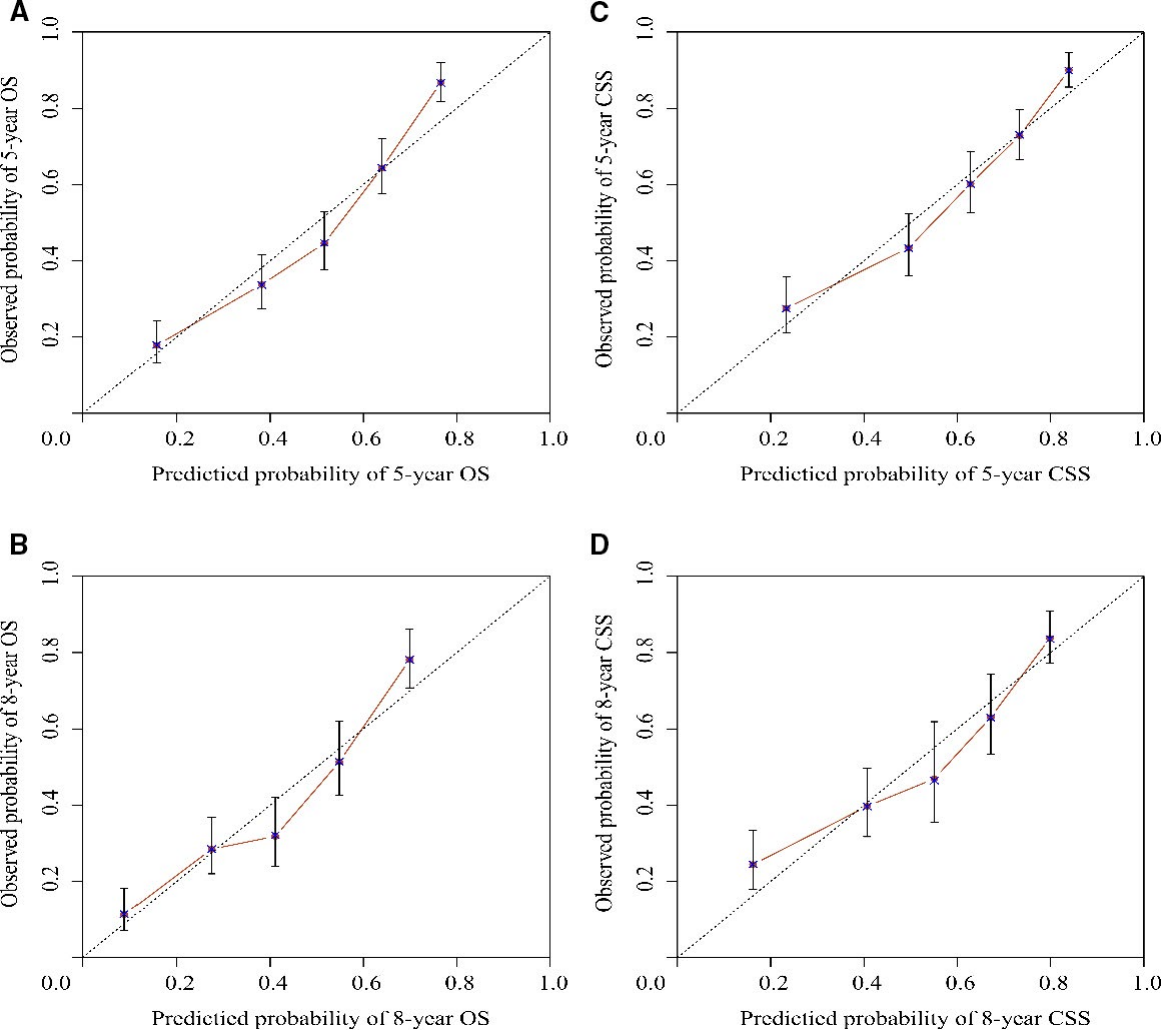
External calibration nomogram for overall survival and cancer‐pecific survival

Internal validation results showed that the C‐indexes were 0.739 and 0.773 regarding OS and CSS. Moreover, the C‐indexes were 0.744 and 0.736 after external validation. The training cohort's AUC values for the OS and CSS were all higher than 0.7, revealing the good specificity and sensitivity of the model (Figure 3). The internal and external calibrations showed that the actual observations were close to the 45° perfect reference line (Figures 4 and 5).

### 3.4 Patient risk stratification

**FIGURE 6:**
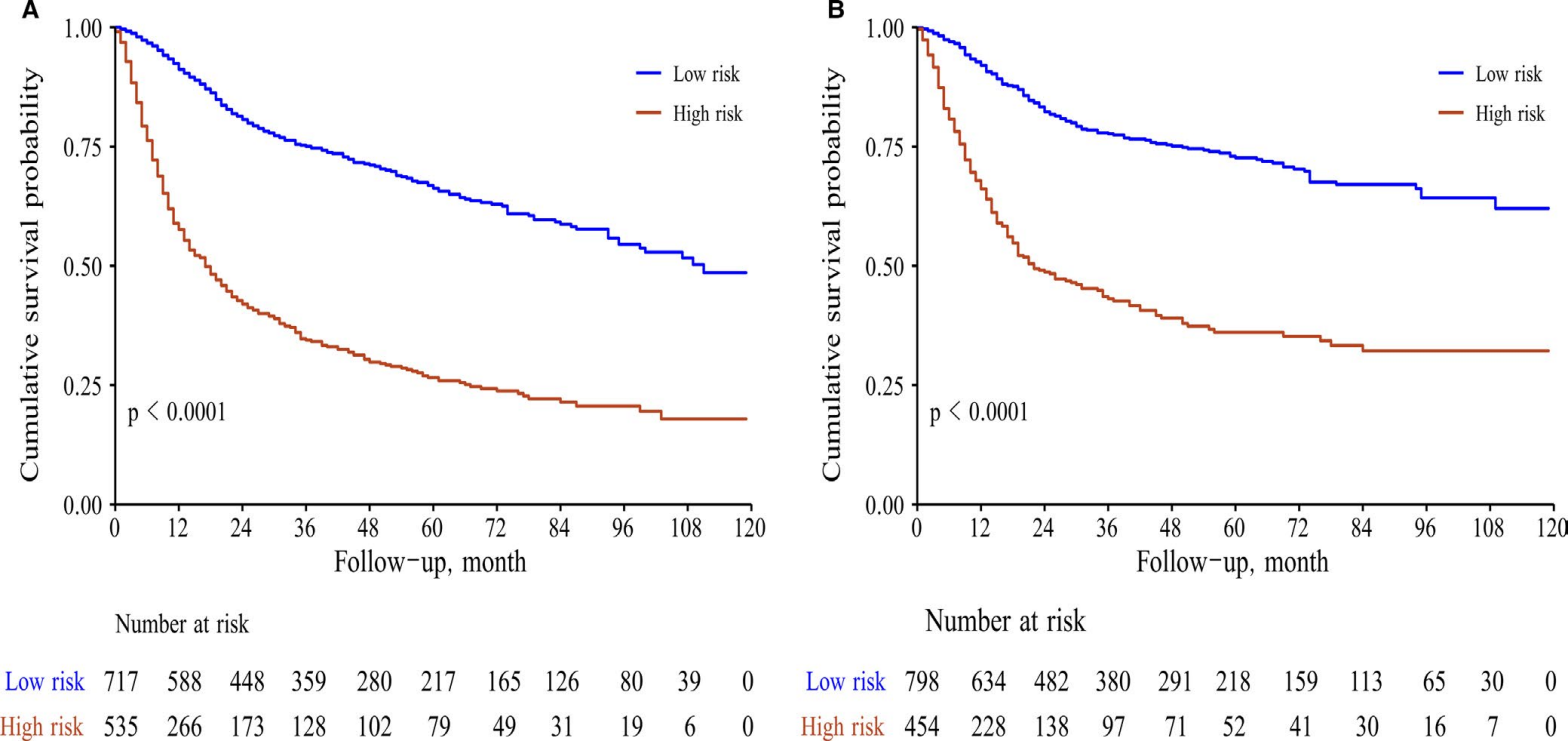
Survival analysis of patients after risk‐stratification (A for overall survival; B for cancer‐specific survival)

We could calculate each patient's total score according to OS and CSS nomograms. Based on the training cohort's OS and CSS nomograms, each patient's total score was calculated, and the cut‐off values were found to be 126 and 184, respectively. Then, we divided the training cohort into high‐ and low‐risk groups based on the cutoff values. After the Kaplan‐Meier OS and CSS analyses and log‐rank tests, the survival curves were drawn. Low‐risk patients’ OS and CSS rates were higher than those of high‐risk patients (*P* < .001) (Figure 6).

## 4 DISCUSSION

According to international epidemiological investigation, GSCC accounts for 25% of OSCC.^19^ Although surgery and other adjuvant treatments have made progress in local tumor control, the mortality rate is still high, and the long‐term survival rate is not optimistic.^20^ To provide a personalized estimate of OS and CSS and risk stratification, we first developed two nomograms to combine the independent risk prognostic factors after survival analysis. Notably, the 8th AJCC manual revealed that in the future version, they would consider the nomogram to conduct patient‐specific prognosis estimates.^21^

We divided total patients into the training and validation groups randomly, which is in accordance with the current research.^22^, ^23^ Moreover, the performances of the nomograms were evaluated via C‐indexes, AUC values and calibration curves. All the C‐indexes and AUC values were higher than 0.7, showing high accuracy. In addition, the calibration curve was in good agreement with the 45° reference line. Cutoff values were obtained after ROC analysis to conduct risk stratification.^24^ Patients assigned to the high‐risk group had a lower survival rate, which was statistically significant (*P* ＜ .05).

Our nomograms consisted of several factors influencing prognosis, which are commonly used in clinical practice. The nomogram showed that younger patients showed favorable OS and CSS (ie, the age group of “15‐45” demonstrated better OS and CSS). In terms of marital status, patients who were married could gain more satisfactory OS and CSS, which was consistent with the research.^24^, ^25^ We found that patients with upper GSCC had better OS and CSS. Mandibular GSCC was more common and prone to invading lymph nodes. The rate of lymph node invasion among the first diagnosis of lower GSCC was 24%‐28%, which is higher than that in the maxillary counterpart.^5^ The survival of patients with GSCC was unsatisfactory due to unilateral and bilateral lymph node metastasis.^26^ Currently, surgery is still an important means to treat GSCC, with a 5‐year survival rate of 50%‐70.4%.^27^ This was in agreement with our results. T, N, and M stages are also the widely used significant factors for constructing nomograms.^28^

The process of predicting long‐term survival by nomograms was simple and practicable. According to individual situations, we selected the subcategories of the independent prognostic factors and drew a vertical line to the point axis. Then, we calculated each subcategory's point together to obtain the predicted values of OS and CSS.^29^ The “rms” package was used to perform this procedure. Notably, the nomogram had advantages over the AJCC TNM staging system. As an example, consider two same‐stage patients with T3N0M0 GSCC: category 1: age: 45, married, grade II, surgery; category 2: age: 40, single, grade III, surgery. The above two categories’ prognosis were the same based on AJCC TNM classification. However, the results were distinct according to the nomogram. The above two patients’ 5‐year OS rates were 75% and 65%, respectively. Moreover, for the two categories of patients, the 5‐year CSS rates were 82% and 75%, accordingly. Thus, the nomogram was of significant importance to guide surgeons and patients to conduct personalized and accurate prognosis predictions.

Our research has apparent advantages and certain drawbacks. First, we conducted a large‐sample and multicenter research in terms of the credible SEER database. Second, for the first time, we reported the construction of nomograms predicting long‐term survival of patients with GSCC throughout the world. Third, after the performance of the nomograms via ROC, C‐index, and calibration curves, our prediction model revealed a high accuracy and sensitivity. However, our study had certain limitations. Related research shows that other relevant factors are significant for the pathogenesis and development of GSCC, such as smoking, alcohol consumption, HPV, and inappropriate oral prosthesis, which were not recruited in the SEER database and thus, were not included our research.^3^, ^4^, ^30^ Hence, we would conduct prospective research to incorporate numerous indicators to establish the prognosis evaluation nomogram model in the future.

In conclusion, we successfully developed two nomograms forecasting 3‐, 5‐ and 8‐year OS and CSS rates on the basis of univariate and multivariate survival analysis. In addition, the performances of the nomograms were warranted. We firmly believe that these nomograms could provide surgeons and patients with personalized prognosis evaluations and could serve as a reference for treatment plan development.

## CONFLICTS OF INTEREST

None.

## AUTHOR CONTRIBUTIONS

Hao Xu and Lei Yan designed this experiment. Lei Yan, Weizhuo Deng and Lina Guan conducted the experiment and analyzed the results and drafted the manuscript under the supervision of Hao Xu. Lei Yan and Hao Xu revised the manuscript finally.

## ETHICAL APPROVAL

The research was approved by the ethical review committee of General Hospital of Xinjiang Military region.

## Data Availability

The data that support the findings of this study are available from the corresponding author upon reasonable request.
